# Reflection of Burnout Severity in a EEG Frequency Pattern

**DOI:** 10.1192/j.eurpsy.2025.1704

**Published:** 2025-08-26

**Authors:** S. Tukaiev, S. Mushta, A. Popov, J. M. A. Ferreira, B. Palamar, M. Makarchuk, I. Zyma

**Affiliations:** 1 Institute of High Technologies, Taras Shevchenko National University of Kyiv; 2 Department of electronic engineering, Igor Sikorsky Kyiv Polytechnic Institute, Kyiv, Ukraine; 3 Faculdade de Medicina, Universidade de Coimbra, Coimbra, Portugal; 4 Department of Social Medicine and Public Health, Bogomolets National Medical University; 5 Institute of Biology and Medicine, Taras Shevchenko National University of Kyiv, Kyiv, Ukraine

## Abstract

**Introduction:**

The burnout develops gradually, unnoticed by the person, and its symptoms may appear after several years and leads to serious mental and behavioral changes. The processes underlying burnout are largely unknown due to the lack of specialized studies aimed at identifying specific biomarkers. Based on this, it is necessary to detect the first, critical moment - the first symptoms of burnout.

**Objectives:**

We aimed to examine the EEG frequencies changes relating to severity of Anxiety Tension stage of Emotional Burnout.

**Methods:**

In this study 752 participants, students and staff of Taras Shevchenko National University of Kyiv (Kyiv, Ukraine) were involved (209 males, mean age = 19.2, 543 females, mean age = 18.28). We used the 84-item Boyko’s Syndrome of Emotional Burnout Inventory to measure the emotional burnout formation. We analyzed separate artefact-free EEG segments in all frequency bands from 0.2 to 45 Hz during resting state (3 min, closed eyes condition). In order to identify the EEG signs of emotional burnout the normalized power spectral densities (PSD) were calculated on the segment from 61 to 70 seconds of recordings.

**Results:**

The revealed burnout-related (Anxiety Tension stage) variables in the spectral characteristics of the EEG characterized by the significant changes in the theta 2 (frontal area and left temporal-parietal cortex), alpha 2 (right parietotemporal cortex) and beta 1 subbands (left frontal-central-right parietal axis).

**Conclusions:**

These data pointed to the influence of Anxiety Tension development mostly on the processes associated with short-term memory and focused attention.

**Disclosure of Interest:**

None Declared

